# Genome Sequence of Bacillus mycoides B38V, a Growth-Promoting Bacterium of Sunflower

**DOI:** 10.1128/genomeA.00245-15

**Published:** 2015-04-02

**Authors:** Adriana Ambrosini, Fernando Hayashi Sant’Anna, Rocheli de Souza, Michele Tadra-Sfeir, Helisson Faoro, Samuel M. Alvarenga, Fabio Oliveira Pedrosa, Emanuel Maltempi Souza, Luciane M. P. Passaglia

**Affiliations:** a Departamento de Genética, Instituto de Biociências, Universidade Federal do Rio Grande do Sul (UFRGS), Porto Alegre, RS, Brazil; b Departamento de Bioquímica e Biologia Molecular, Universidade Federal do Paraná (UFPR), Centro Politécnico, Curitiba, PR, Brazil; c Fundação Estadual de Pesquisa Agropecuária (FEPAGRO), Porto Alegre, RS, Brazil

## Abstract

Bacillus mycoides B38V is a bacterium isolated from the sunflower rhizosphere that is able to promote plant growth and N uptake. The genome of the isolate has approximately 5.80 Mb and presents sequence codifiers for plant growth-promoting characteristics, such as nitrate reduction and ammonification and iron-siderophore uptake.

## GENOME ANNOUNCEMENT

Bacillus mycoides is a sporogenic Gram-positive bacterium belonging to the B. cereus group (1, 2). Some environmental isolates of B. mycoides are able to induce systemic resistance (3) and promote plant growth (4). The B38V strain was isolated from the rhizosphere of the sunflower (Helianthus annuus L., cultured in Rio Grande do Sul, Brazil, 28°30′43″S, 50°56′02″W) using N-free and semisolid culture medium and anaerobic conditions. The B38V strain has the potential to promote sunflower growth and to contribute to NPK content (A. Ambrosini, T. Stefanski, B. Lisboa, A. Beneduzi, L. Vargas, and L. Passaglia, submitted for publication). 16S rRNA gene sequencing and the presence of rhizoidal colonies were used for the identification of the isolate, which presented counter-clockwise curved radial filaments (1, 5).

The genomic sequence of the B38V strain was accessed to explore its potential effectiveness as an agricultural inoculant, particularly regarding the genes related to nitrogen turnover in soil. Genomic DNA was extracted (6) and constructed into a 500- to 1,200-bp insert library, and the sequencing was performed at MiSeq Illumina platforms using the MiSeq Regent kit v2. A total of 601,358 paired-end reads were generated, with an average length of 240 bp and approximately 41-fold coverage. The assembly was performed with four different software packages, A5-miseq (7), CISA (8), CLC Genomics Workbench (http://www.clcbio.com/products/clc-genomics-workbench/), and SPADES (9); A5-miseq was chosen due to the lower value of *N_50_* (149,872), fewer contigs (138), completeness of 99.43%, and an absence of contamination (10). A total of 106 scaffolds were submitted to the RAST server (11) to obtain automatic annotation.

The genome of B. mycoides strain B38V comprises 5,784,959 bp, with an average G+C content of 35.20%. A total of 5,933 coding DNA sequences and 97 tRNA, 1 16S rRNA, 1 23S rRNA, and 14 5S rRNA genes were predicted. Several genes were related to the siderophore transport system and biosynthesis. Despite their nitrogen-fixing ability, the genes related to this process were not found. The genome of the B38V strain also contained several genes related to complete denitrification, such as respiratory nitrate reductase (*narGHIJ*), nitrite reductase (*nirK*), nitric oxide reductase (*norBC*), and nitrous oxide reductase (*nosZ*). Genes related to nitrate and nitrite ammonification were also found, such as *nrfBCDEFGHX*. Future studies may provide insight into the genomic basis of plant growth-promotion characteristics, focusing on nitrogen metabolism and the respective traits related to plant development and health.

### Nucleotide sequence accession numbers.

This whole-genome shotgun project has been deposited at GenBank under the accession no. JYFS00000000. The version described in this paper is the first version, JYFS01000000.

## References

[1] Turchi L, Santini T, Beccari E, Di Franco C 2012 Localization of new peptidoglycan at poles in *Bacillus mycoides*, a member of the *Bacillus cereus* group. Arch Microbiol 194:887–892. doi:10.1007/s00203-012-0830-1. 22773111 PMC3445799

[2] Jiménez G, Urdiain M, Cifuentes A, López-López A, Blanch AR, Tamames J, Kämpfer P, Kolstø AB, Ramón D, Martínez JF, Codoñer FM, Rosselló-Móra R 2013 Description of *Bacillus toyonensis* sp. nov., a novel species of the *Bacillus cereus* group, and pairwise genome comparisons of the species of the group by means of ANI calculations. Syst Appl Microbiol 36:383–391. doi:10.1016/j.syapm.2013.04.008. 23791203

[3] Kloepper JW, Ryu CM, Zhang S 2004 Induced systemic resistance and promotion of plant growth by *Bacillus* spp. Phytopathology 94:1259–1266. doi:10.1094/PHYTO.2004.94.11.1259. 18944464

[4] Karagöz K, Ateş F, Karagöz H, Kotan R, Çakmakçı R 2012 Characterization of plant growth-promoting traits of bacteria isolated from the rhizosphere of grapevine grown in alkaline and acidic soils. Eur J Soil Biol 50:144–150. doi:10.1016/j.ejsobi.2012.01.007.

[5] Di Franco C, Beccari E, Santini T, Pisaneschi G, Tecce G 2002 Colony shape as a genetic trait in the pattern-forming *Bacillus mycoides*. BMC Microbiol 2:33. doi:10.1186/1471-2180-2-33. 12429070 PMC138795

[6] Ambrosini A, Beneduzi A, Stefanski T, Pinheiro FG, Vargas LK, Passaglia LMP 2012 Screening of plant growth promoting rhizobacteria isolated from sunflower (*Helianthus annuus* L.). Plant Soil 356:245–264. doi:10.1007/s11104-011-1079-1.

[7] Coil D, Jospin G, Darling AE 2015 A5-miseq: an updated pipeline to assemble microbial genomes from Illumina MiSeq data. Bioinformatics 31:587–589. doi:10.1093/bioinformatics/btu661. 25338718

[8] Lin SH, Liao YC 2013 CISA: contig integrator for sequence assembly of bacterial genomes. PLoS One 8:e60843. doi:10.1371/journal.pone.0060843. 23556006 PMC3610655

[9] Bankevich A, Nurk S, Antipov D, Gurevich AA, Dvorkin M, Kulikov AS, Lesin VM, Nikolenko SI, Pham S, Prjibelski AD, Pyshkin AV, Sirotkin AV, Vyahhi N, Tesler G, Alekseyev MA, Pevzner PA 2012 SPAdes: a new genome assembly algorithm and its applications to single-cell sequencing. J Comput Biol 19:455–477. doi:10.1089/cmb.2012.0021. 22506599 PMC3342519

[10] Parks DH, Imelfort M, Skennerton CT, Hugenholtz P, Tyson GW 2014 CheckM: assessing the quality of microbial genomes recovered from isolates, single cells and metagenomes. PeerJ PrePrints 2:e554v1. doi:10.7287/peerj.preprints.554v1. PMC4484387 25977477

[11] Aziz RK, Bartels D, Best AA, DeJongh M, Disz T, Edwards RA, Formsma K, Gerdes S, Glass EM, Kubal M, Meyer F, Olsen GJ, Olson R, Osterman AL, Overbeek RA, McNeil LK, Paarmann D, Paczian T, Parrello B, Pusch GD, Reich C, Stevens R, Vassieva O, Vonstein V, Wilke A, Zagnitko O 2008 The RAST server: Rapid Annotations using Subsystems Technology. BMC Genomics 9:75. doi:10.1186/1471-2164-9-75. 18261238 PMC2265698

